# Outcomes of hamstring graft with preserved tibial insertion for ACL reconstruction: systematic review and meta-analysis

**DOI:** 10.1007/s00590-023-03698-5

**Published:** 2023-08-29

**Authors:** Nicolas Vari, Etienne Cavaignac, Marie Cavaignac, Émilie Bérard, Vincent Marot

**Affiliations:** 1 Musculoskeletal Institute, Hôpital Pierre Paul Riquet, CHU Toulouse, Toulouse, France; 2 Clinique Rive Gauche, Toulouse, France; 3 Department of Epidemiology, Health Economics and Public Health, UMR 1295 CERPOP, University of Toulouse, INSERM, UPS, Toulouse University Hospital (CHU), Toulouse, France; 4 Orthopaedics Unit, Hospital Nostra Senyora de Meritxell, Escaldes-Engordany, Andorra

**Keywords:** ACL reconstruction, Hamstring graft, Pedicled, Non-detached

## Abstract

**Purpose:**

Evaluate the outcomes of ACL (Anterior Cruciate Ligament) reconstruction techniques that use a hamstring graft with a preserved tibial insertion and compare them to standard techniques.

**Methods:**

A systematic literature review and meta-analysis was done of the PubMed, MEDLINE, Cochrane and Ovid databases to identify published clinical studies on ACL reconstruction in which a non-detached hamstring tendon (NDHT) was used as a graft and to compare them to studies in which a detached hamstring tendon (DHT) or other techniques were used. The eligible studies were analyzed for the knee laxity, Lachman test, pivot shift test, joint range of motion, anterior drawer, pain, re-tear, revision surgery, Lysholm score, Tegner score, ACL-RSI scale, KOOS, IKDC, SNQ and Howell scale.

**Results:**

Twelve articles in which NDHT was used for ACL reconstruction were analyzed. There was no significant difference between NDHT and DHT in the Lachman > 1 (*p* = .07), pivot shift test > 1 (*p* = .40), re-tears (*p* = .62), pain (*p* = .85) and the Tegner score (*p* = .95). However, the outcomes were somewhat better with the NDHT technique for the Lachman (*RR* = 0.30; 95% CI 0.08–1.12), pivot shift test (*RR* = 0.50; 95% CI 0.10–2.49) and re-tears (*RR* = 0.66; 95% CI 0.13–3.42). The other criteria were not included in the meta-analysis because of lack of data or because specific outcome scores were used in each article.

**Conclusion:**

NDHT techniques provide similar results to DHT for ACL reconstruction and tend to produce better stability and a lower re-tear rate.

**Supplementary information:**

The online version contains supplementary material available at 10.1007/s00590-023-03698-5.

## Introduction

Anterior cruciate ligament (ACL) tears are one of the most common knee injuries [1–4]. Several types of grafts have been used for reconstruction surgery to restore knee stability [5–8]. Among the available reconstruction techniques, those using the hamstring tendon are among the most common and produce very good results [4, 7–9]. Standard techniques use the gracilis (G) and semitendinosus (ST) which are harvested and detached from their tibial insertion [10–13] (detached hamstring graft, DHT).

Some surgeons have proposed using a graft in which the tibial insertion of the hamstring tendons is preserved [10, 12, 13] (non-detached hamstring graft, NDHT). They contend that preserving the tibial insertion helps to retain the graft’s innervation and vascularization. Zaffagnini et al. [14] showed that a well-defined vascular network and a wide swath of nerve fibers were present on the insertion of these tendons. Furthermore, the construct is more solid due to the double tibial fixation (original insertion + mechanical fixation) [15–17]. The goal of using this technique is to achieve better graft integration [18–21, 21–23] in order to attain better functional outcomes and a lower retear rate.

To date, the only literature review on this subject was published by Ruffili et al. [24] in 2015. They included six studies with a total of 363 patients but could not group them or compare their outcomes. Nevertheless, three of the studies found that the function outcome scores were better when the tibial insertion of the hamstring grafts was preserved. The three other studies did not find a statistically significant difference. Thus, it seems appropriate to carry out of an updated systematic literature review and meta-analysis of all the published results with these new techniques in which the tibial insertion of the hamstring tendons is preserved. The analysis soughs to answer the following question: In ACL reconstruction, does using a NDHT graft produce clinical, functional and imaging outcomes that are at least equal to using a DHT graft?

## Methods

### Study eligibility (inclusion and exclusion criteria)

Eligible studies were prospective clinical trials reporting the results of NDHT and those comparing NDHT with DHT. The ineligible criteria were: reviews and articles published in languages other than French or English, studies with less than 12-month follow-up, studies investigating the outcomes of multi-ligament surgery and retrospective studies. We chose to exclude retrospective studies because their scientific value is less than that of prospective studies.

### Literature search

An exhaustive search of articles published in the PubMed, MEDLINE, Cochrane and Ovid databases was done by following PRISMA guidelines [25]. The following keywords were used in the search: “non-detached”, “preserved tibial insertion” in combination with the term “ACL reconstruction”. All articles published up to January 1, 2022 were included, including articles published online. The references of the primary and review articles and main orthopedic textbooks were cross-referenced to identify any additional articles meeting the inclusion criteria that had not been identified during the primary search.

### Study selection and data abstraction

The articles identified in the searches were screened by two authors who extracted the data independently; any conflicts were resolved before the final analysis. In clinical and comparative studies, the endpoints were compiled. When the data needed for a statistical analysis were not presented, the study authors were contacted by email. The following data were extracted from each study: (1) knee stability, including the mean side-to-side difference and the percentage of side-to-side difference above 3 mm (using a KT-1000/2000 arthrometer or Rolimeter), Lachman test graded as normal (score 1) or abnormal (score 2 or 3), pivot shift test graded as normal (grade 0 or 1) or abnormal (grade 2 or 3), joint range of motion, anterior drawer, pain, retear and revision surgery; (2) functional outcome scores, including the Lysholm, Tegner, ACL-RSI, KOOS and IKDC; (3) MRI criteria such as the signal-to-noise quotient (SNQ), Howell score and scores specific to each study. Only studies that were selected by the two authors were included in this meta-analysis.

### Data analysis

The primary outcome measures were the clinical, functional and MRI parameters listed above. Study results were tabulated as the number of events and total number of subjects in the experimental (NDHT) versus control (DHT) groups for Lachman = 2 or 3, pivot shift = 2 or 3, pain and re-tear. Study results were tabulated using the mean and standard deviation (SD) together with the total number of subjects in the experimental versus control groups for continuous endpoints (Tegner score). Missing mean and SD were assessed from the median, range, and the size of the sample [26]. Missing SD (without median and range) was assessed according to the sample size and means from reported *P* values [27].

To describe the experimental results (NDHT), the frequency of an event or the weighted mean (continuous endpoints) was calculated together with 95% confidence intervals (95%CI). The risk ratio of an event in the experimental versus control group was calculated according to the inverse variance approach with their 95% CI. When the number of events was equal to zero in each group, it was imputed to one to estimate the risk ratio. The mean differences between experimental and control group were estimated for continuous endpoints according to the inverse variance approach with their 95% CI.

Forest plots were used to assess heterogeneity across studies, as well as Cochran’s heterogeneity statistic and Higgins I^2^ coefficients [28]. A *P* value < 0.1 or *I*^2^ > 50% was considered suggestive of statistical heterogeneity, prompting random effects modeling. To assess the risk of bias, the level of evidence of each study included in this meta-analysis was determined and included in the summary table (online Appendix). Funnel plots were generated to assess small-study effects [29]. The Review Manager 5.2 analysis software (The Cochrane Collaboration, Copenhagen, Denmark) was used to perform these analyses.

## Results

### Literature search, study selection and characteristics

**Fig. 1:**
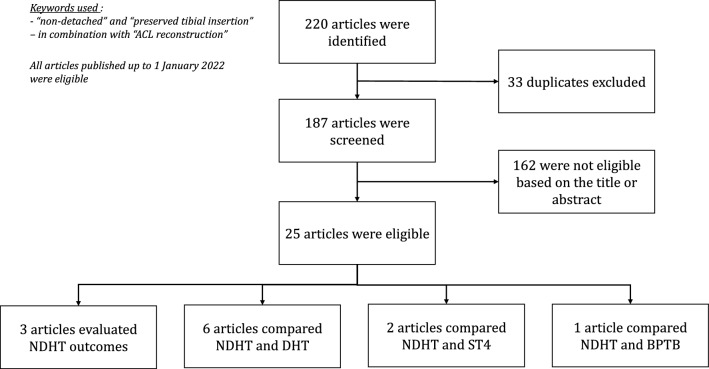
Flow chart summarizing the selection of articles according to PRISMA guidelines. *NDHT* non-detached hamstring tendon graft*, DHT* Detached hamstring tendon graft*, ST4* four-bundle semitendinosus graft*, BPTB:* Bone patellar tendon bone graft

The literature search of the various databases identified 220 articles. After excluding duplicates, 187 articles remained. After screening the title and abstract of these articles, 25 articles were retained, and the full text read to evaluate their eligibility. In the end, 12 articles met the inclusion criteria: 3 studies reported the results of NDHT [30–32], 6 studies compared the results of NDHT and DHT [33–38], 3 studies compared the results of NDHT and other ACL reconstruction techniques (2 used the ST4 technique [37, 39] and 1 used a bone-patellar tendon-bone graft [40]). (Fig. 1) The main features of the selected studies are summarized in the online Appendix.

### Analysis of NDHT outcomes

#### Primary endpoints

The meta-analysis of NDHT outcomes found stable knees with good clinical scores and few complications (Table 1).

**Table 1:** Outcomes of ACL reconstruction with NDHT

	No. of studies	No. of patients	Mean* or % (95% CI)
KT1000 Arthrometer (a)*	3	153	2.61 (2.48–2.74)
Lachman > 1	4	148	4.73 (1.31–8.15)
Pivot shift > 1	6	206	0.49 (0.00–1.43)
Anterior drawer	2	104	2.88 (0.00–6.10)
Pain	4	156	10.90 (6.01–15.79)
Retear	5	174	0.00 (0.00–0.00)
Surgical revision	3	131	5.34 (1.49–9.19)
Other complications	3	173	7.51 (0.00–11.44)
Tegner score (b)*	9	152	6.60 (6.32–6.88)

*Mean values given; No. of patients is the total of patients where the criteria was evaluated

No.: Numbers; NDHT: Non-detached hamstring tendon graft; CI, Confidence interval

(a)Values shown are in millimeters

(b)Values shown are in points

#### Other endpoints

The meta-analysis could not be completed for the other endpoints: (1) 3 articles incorporated KT2000 Arthrometer measurements but did not disclose the SD (2) none of the articles evaluated the knee ROM and the Lysholm had no mean ± SD values; (3) none of the articles evaluated the ACL-RSI, KOOS and TTE; (4) the IKDC was evaluated in a single article; (5) 3 articles evaluated the SNQ but did not disclose the SD; (6) only one article measured the Howell grade and (7) the specific scores used in each study could not be combined.

### Analysis of outcomes of NDHT versus DHT

#### Primary endpoints

**Fig. 2:**
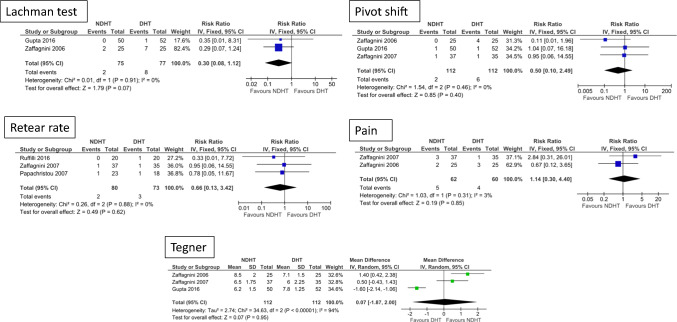
Forest plots comparing the Lachman, pivot shift, retear rate, pain and Tegner score between the NDHT (non-detached hamstring tendon) and DHT (detached hamstring tendon) techniques for ACL reconstruction

There was no significant difference between NDHT and DHT in the Lachman > 1 (*p* = 0.07), pivot shift test > 1 (*p* = 0.40), re-tears (*p* = 0.62), pain (*p* = 0.85) and the Tegner score (*p* = 0.95) (Fig. 2). However, the outcomes were somewhat better with the NDHT technique for the Lachman (*RR* = 0.30; 95% CI 0.08–1.12), pivot shift test (*RR* = 0.50; 95% CI 0.10–2.49) and re-tears (*RR* = 0.66; 95% CI 0.13–3.42). There was considerable heterogeneity in the analysis of the Tegner score since studies go in the opposite direction.

#### Other endpoints

The meta-analysis could not be completed on the other endpoints: (1) some of the criteria were analyzed in one study only (KT2000 Arthrometer, ROM, anterior drawer, re-operation, other complications, IKDC, Howell) (2) two studies evaluated the Lysholm score but did not disclose the SD; (3) none of the articles evaluated the ACL-RSI, KOOS and TTE (Tibial Tunnel Enlargement); (4) 3 articles evaluated the KT1000 and SNQ but did not disclose the SD; (5) the specific scores used in each study could not be combined.

#### Funnel plots

No publication bias was identified by visual inspection of the funnel plots (online appendix).

## Discussion

The most important findings of the present study were that techniques using NDHT grafts produce outcomes that are at least equivalent to techniques using DHT grafts for ACL reconstruction. The analysis of outcomes with NDHT found stable knees with good clinical scores and few complications. A comparison of the NDHT and DHT techniques found similar results in terms of stability, pain and function.

Our meta-analysis findings are consistent with the review of literature published by in 2015 by Ruffilli et al. [24] of 363 patients. Our analysis of studies using the NDHT techniques found very good clinical and functional outcomes, particularly for a positive pivot shift test (0.49%; 95% CI, 0.00–1.43%) and retear rate (0.00%; 95% CI, 0.00–0.00%). These findings are also consistent with retrospective studies that were not included in this meta-analysis. Buda et al. [16], in their retrospective study of 28 patients, found excellent results in the functional IKDC with a mean score of 93.8/100 and in the MRI results with 25/28 patients having a Yamato score of 1. Meynard et al. [41] did a retrospective study of long-term outcomes in patients who had undergone ACL reconstruction with NDHT and had a mean follow-up of 9.9 years. The mean KOOS was 86.3 ± 16.3, 94.4% had a negative pivot shift and 49/50 patients had a knee that was labelled as “normal” or “nearly normal” during the objective IKDC evaluation (Grade A or B).

In our meta-analysis, the results of the Lachman (RR = 0.30, 95% CI 0.08–1.12), pivot shift test (*RR* = 0.50, 95% CI 0.10–2.49) and retear rate (RR = 0.66, 95% CI 0.13–3.42) were not statistically significant but were better when using the NDHT technique. A lack of statistical power may have contributed to this lack of statistical significance since the meta-analysis was based on only two or three studies for each of these criteria.

The results of the pain assessment in our meta-analysis (*RR* = 1.14, 95% CI 0.30–4.40) were slightly better for the DHT group. We can't find an explanation for these results because the only difference between the two techniques is that the tibial insertion of the hamstring tendons is detached in one group (DHT) but not the other (NDHT).

The graft’s signal in MRI is now widely used to evaluate good graft incorporation and to validate certain surgical techniques [42, 43]. Grassi et al. [33] used MRI as the endpoint in their study. MRI was done at 4 and 18 months postoperative to compare a NDHT technique to a conventional DHT technique. The signal was lower in the group in which the tendon’s tibial insertion was preserved at 4 months (*p* = 0.008) and 18 months (*p* = 0.028), suggestive of better graft ligamentization.

In our systematic literature review, no retears occurred in the 174 patients who underwent ACL reconstruction with NDHT graft (0.00; 95% CI 0.00%–0.00%). The absence of retears can be explained by the relative short duration of the follow-up in the studies included. In fact, other than the studies by Marcacci et al.[31] with an 11-year follow-up and by Zaffagnini et al. [40] with an 8-year follow-up, the other studies had a mean follow-up of about 2 years. It would be interesting to evaluate the retear rate in the longer term, when patients have returned to their sports activity. This may confirm that the NDHT construct is indeed more solid.

### Limitations

The current study has several limitations. First, not every article included featured a randomized controlled trial (online Appendix); this can bring about differences in the populations studied, which can alter the results obtained. Second, the criteria studied in this meta-analysis could only be compared directly in a limited number of studies, either because the criteria were not the same or because some data were missing. Nevertheless, this is the only meta-analysis so far to analyze a large cohort of patients who have undergone ACL reconstruction with NDHT graft. Third, while the surgical techniques use the same tendons (ST and G), there are differences between them, particularly in the fixation method used (screw, Endobutton, staple), which can introduce bias in the results.

## Conclusions

NDHT techniques provide similar results to DHT for ACL reconstruction and tend to produce better stability and a lower re-tear rate.

### Supplementary information

Below is the link to the electronic supplementary material.

Supplementary file1 (DOCX 101 KB)
