# Supplementary material for: Outcomes of hamstring graft with preserved tibial insertion for ACL reconstruction: systematic review and meta-analysis
Source: Eur J Orthop Surg Traumatol. 2023 Aug 29;34(1):67–73. doi: 10.1007/s00590-023-03698-5 (PMC10771374; doi:10.1007/s00590-023-03698-5)
Supplement: Supplementary file 1 — Supplementary file1 (DOCX 101 KB) [file 590_2023_3698_MOESM1_ESM.docx]

**Appendix**

**Summary of prospective studies evaluating non-detached hamstring grafts in anterior cruciate ligament reconstruction**

| **Article** | **Design / Level of evidence** | **Mean follow-up (months)** | **Surgery** | | **Outcomes** |
| --- | --- | --- | --- | --- | --- |
|  |  |  | **Control group (DHT) / n (patients)** | **Test group (NDHT) / n (patients)** |  |
| ***Grassi et al.***  ***2020*** | RCT / 4 | 18 | STG / 10 | STG / 10 | SNQ, Howell  Tunnel + graft score |
| ***Zhang et al.***  ***2020*** | RCT / 2 | 60 | STG / 19 | STG / 18 | KT-1000 Arthrometer  Lysholm, Tegner, IKDC, SNQ |
| ***Bahlau et al.***  ***2019*** | PC / 2 | 30 | X | STG / 24 | KT-1000 Arthrometer, IKDC  Lachman, ROM°, Lysholm |
| ***Liu et al.***  ***2018*** | RCT / 2 | 24 | STG / 19 | STG / 18 | KT-1000 Arthrometer  Lysholm, Tegner, IKDC, SNQ |
| ***Sinha et al.***  ***2018*** | PC / 3 | 24 | X | STG / 79 | KT-1000 Arthrometer  ROM°, Other complications  Lysholm, Tegner |
| ***Ruffilli et al.***  ***2016*** | RCT / 2 | 24 | ST4 / 20 | STG / 20 | Re-tear, Tegner, IKDC  Ligamentization score |
| ***Gupta et al.***  ***2016*** | RCT / 1 | 24 | STG / 52 | STG / 50 | KT-1000 Arthrometer  Lachman, Pivot shift, Tegner  Anterior tibial translation |
| ***Zaffagnini et al.***  ***2010*** | RCT / 2 | 96 | BPTB / 39 | STG / 40 | KT-2000 Arthrometer  Pivot shift, Pain, Tegner,  Re-tear, Re-operation  Other complications, IKDC |
| ***Marcacci et al.***  ***2009*** | CS / 4 | 132 | X | STG / 54 | KT-2000 Arthrometer  Lachman, Pivot shift, ROM°, Pain, Re-tear, Re-operation  Anterior tibial translation  Lysholm, Tegner  Other complications |
| ***Zaffagnini et al.***  ***2007*** | RCT / 2 | 46.8 | STG / 35 | STG / 37 | KT-2000 Arthrometer  Pivot shift, ROM°, Pain  Re-tear, Re-operation, Tegner |
| ***Papachristou et al.***  ***2007*** | PCC / 3 | 19.05 | STG / 18 | STG / 23 | Pivot shift test, Re-tear, IKDC |
| ***Zaffagnini et al.***  ***2006*** | PCC / 3 | 60 | ST4 / 25  BPTB / 25 | STG / 25 | Lachman, Pivot shift  Pain, Tegner, IKDC |

*DHT, detached hamstring tendon ; NDHT, non-detached hamstring tendon ; RCT, randomized controlled trial ; PC, prospective cohort ; CS, case series ; PCC, prospective comparative cohort ; STG, semitendinosus-gracilis ; ST4, quadrupled semitendinosus ; BPTB, bone patellar tendon bone ; SNQ, signal to noise quotient ; IKDC, International knee documentation committee score ; ROM°, range of motion in degree*

**Funnel plots**

**Funnel Plot - Lachman**

**Funnel Plot - Pivot Shift Test**

**Funnel Plot - Re-tear rate**

**Funnel Plot - Pain**

**Funnel Plot - Tegner**
